# Annotations

**Published:** 1888-08-18

**Authors:** 


### Annotations.

**The British Medical Association.**

The recent meeting of the British Medical Association at Glasgow ought to form a recollection equally satisfactory to the members of the association, and to the inhabitants of the second city of the Empire. St. Mungo's City has proved that, amid the noise of machinery and the clinking of gold, it can lend an ear to the grave thoughts of science, and the wise utterances of the professors of the healing art. This is not a place to enter into any analysis of the subjects discussed, or the merits of those who contributed to the discussion; these are matters for purely medical journals to take up and deal with. But it is perfectly fitting to note, in the cordial welcome accorded by the great busy city of the north to the doctors who gathered there from far and near, for the help which mutual intercourse can give, the growing appreciation felt by the public for medical and sanitary science. Much of this is due to the open and frank attitude of medical men. The physician is no longer related to the sorcerer, nor his science to the black arts. He can give a reason for every step he takes; his diagnosis is not guess work. nor his treatment quackery. He knows what he is about; and this is a thing which men, who in other walks of life know what *they* are about, never fail to respect. It must be admitted that Scotland is a very medical country; it has more than one famous factory for the turning-out of fully equipped doctors, and the interest in things medical is exceptionally general and widespread. Glasgow has proved that the canny Scot has a taste for physic as well as for theology, and is perhaps developing a growing fancy for sanitation. But the long and full reports of the meetings given in all the important newspapers of the country, the fact that the chief London journals have each had a leader on the subject, show to what an extent the British Medical Association is a power in the land. Indeed, a close-set phalanx of 12,000 thoughtful, intelligent, and energetic men—and to this number the membership of the British Medical Association has now reached—is a fact that cannot be ignored. The chronicle of their sayings and doings may not be as sensational as the account of a battle, but there is no doubt that people are more and more feeling the importance of that quiet and unpretending army whose motto is "Our duty is to save." There they stand—no, they do not stand, they go on, adding to their number as they go—a piece of leaven to leaven the whole lump of our population with respect for the laws of health.

**The Women's Jubilee Offering.**

The committee appointed by the Queen in June last to co operate with the Duke of Westminster, Sir James Paget, and Sir Rutherford Alcock, in organising a scheme for the nursing of the sick poor at their homes have now finished their labours. They have arranged for the restoration of St. Katharine's Hospital to the uses for which it was originally intended, and in connexion with this institution have formed central committees and training-schools in the capital cities of each section of the kingdom. It must be admitted that on the face of things the committee do not seem to have cut their coat according to their cloth. £70,000 seems a large sum, and so it is for a private fortune. It is a fair, though not a large endowment, for a single institution; but it forms a very small basis for the weaving of a mantle of charity that shall spread over three kingdoms. The Jubilee offering can be regarded only as the nucleus of a great fund—we wish it could become as large as the National Debt—which will one day provide so many skilled nurses that no cottar in the farthest Highland glen, no worn-out labourer in the dreariest slum, need die for lack of proper help and care. This is still a dream of the future, but the first step has been taken, and our vision will not fail to be realised if the Queen's subjects will follow the example of their Sovereign, and give in proportion to their means for the help of those who are in need. One suggestion alone we would make. The capital sum of £72,588 has been invested in Government stock, paying two and a-half per cent. This is a needless waste of the committee's resources. Government stocks are undoubtedly secure, but there are many investments practically as safe which would yield interest at the rate of four per cent. This would make a great difference to the income of the fund, which at present amounts to a little over £2,000 per annum. We do not doubt that the committee have been guided in their choice of investments by prudent considerations; but to thus sacrifice nearly £1,200 a-year of their available income is neither necessary nor expedient. Money subscribed for so good a cause should not, indeed, be wasted in risky speculations; but, on the other hand, it is the duty of those who administer it to see that as high a rate of interest is obtained as is compatible with perfect security.

**A Storm at Owen's College.**

There has been a rather warm controversy in the Manchester papers concerning the recent appointment to the chair of surgery at the Victoria University, better known as Owens College. The authorities, after what we may presume has been a carefully-conducted inquiry, have appointed Mr. A. W. Hare; and this appointment has given a good deal of dissatisfaction, more particularly to the students attending the medical classes. It is proper to point out that if Mr. Hare's appointment had given universal satisfaction it would probably have been the first appointment ever made to any chair whatever which had been so fortunate. No man was ever yet made Prime Minister or parish beadle but some better man was passed over, at any rate, according to the judgment of his friends. There are, however, certain features in the case which are worthy of a little consideration. A few of the more ignorant and inexperienced critics have said that as Mr. Hare's higher qualifications are marked with the Edinburgh stamp, whilst his principal opponent's bear the London impress, therefore Mr. Hare's qualifications are of a somewhat lower academic value than his rival's. That, of course, is mere schoolboy nonsense, and would never have been advanced by any man possessed of experience and knowledge of the world. A fact of real importance is that whilst Mr. Hare has only been fully qualified for six years, his principal opponent has been qualified for eighteen. He has besides held the appointments of consulting surgeon to a Manchester hospital, and lecturer in surgery at the very university which has passed him over in favour of a younger man. This apparent disregard of service rendered is one of the chief grounds of dissatisfaction to a number of persons who have a deep interest in the appointment. It would be presumption on our part to question the wisdom of the decision arrived at by the authorities. To us the mere external and obvious facts are known; to them all the circumstances necessary to the forming of a sound and honest judgment. Whilst therefore it may appear on the first blush that the appointment has been made without due regard to the merits of the case, there may be facts known to the responsible authorities which amply justify the turning of the scale in the direction determined by them. Unless there has been a real and flagrant departure from the obvious principles which should govern such appointments, we counsel the non-contents to accept the facts with equanimity, and to await the verdict of results. It is almost impossible to believe that a governing body, placed in so responsible and critical a position as that of the authorities at Owens College, would act otherwise than according to the dictates of honour and justice, and with a single eye to the welfare of the University.

